# An Overview of the Research Status and Advances in Precision Feeding Technology and Equipment in Aquaculture

**DOI:** 10.3390/ani16121898

**Published:** 2026-06-18

**Authors:** Ke Chen, Sixian Li, Tieli Lyu, Dongfang Li, Zhiqiang Zhou, Jieyu Xian, Maohua Xiao

**Affiliations:** 1 College of Engineering, Nanjing Agricultural University, Nanjing 210031, China; lisixian@stu.njau.edu.cn (S.L.); 2024212019@stu.njau.edu.cn (T.L.); dongfangli@njau.edu.cn (D.L.); xianjy@njau.edu.cn (J.X.); 2 Agricultural Mechanization Technology Promotion Station of Kunshan, Kunshan 215330, China; zzq.9001@163.com

**Keywords:** precision feeding, aquaculture, feeding equipment, machine vision, feeding decision-making

## Abstract

Precision feeding is an important foundation for improving production efficiency in aquaculture, reducing feed waste, mitigating water pollution, and promoting the intelligent development of aquaculture. Conventional feeding practices remain heavily dependent on operator experience and are typically executed at predetermined times or fixed ration levels. Such approaches frequently result in extensive feeding management, poor adaptability, low feed utilization efficiency, and delayed responses to environmental changes. Advances in machine vision, the Internet of Things, machine learning, deep learning, and automatic control have progressively shifted aquaculture feeding research beyond standalone automatic feeders toward integrated systems encompassing demand perception, intelligent decision-making, precise control, and equipment coordination. This paper reviews the state of the art in precision feeding technologies and equipment in aquaculture. At the technical level, it summarizes advances in feeding demand perception, intelligent feeding decision-making, and precise control and execution. At the equipment level, it reviews the main types, design features, and field application status of precision feeding equipment in intensive aquaculture, pond aquaculture, and offshore aquaculture scenarios. Despite the considerable progress achieved, the practical deployment of precision feeding still faces several limitations. Environmental disturbances, water turbidity, illumination variation, and sensor drift may compromise the reliability of feeding demand perception. Existing decision-making models frequently exhibit limited generalizability across species, growth stages, and aquaculture scenarios. Moreover, insufficient integration of sensing, decision-making, and execution restricts the development of fully closed-loop feeding systems. High initial investment, maintenance costs, and the shortage of skilled personnel further constrain the adoption of precision feeding equipment, particularly in resource-limited regions. On this basis, the main challenges including sensing accuracy, model practicability, closed-loop control, equipment reliability, and standardization, are examined. Future development trends are also discussed, covering multi-source information fusion, synergy between mechanistic models and data-driven methods, system-level closed-loop control, equipment modularization, and industrial application. This review is expected to provide a reference for subsequent research and engineering applications.

## Simple Summary

Feeding fish and shrimp is a critical task in aquaculture. Traditionally, farmers feed by hand or use simple timers, which often results in either too little food (—slowing animal growth—). Scientists have been developing smarter ways to decide when, where, and how much to feed. These new approaches use cameras to watch how fish behave, sensors to track water conditions, and computer programs to make feeding decisions automatically. At the same time, new types of feeding machines—ranging from rail-guided robots to autonomous boats—have been built to deliver feed more precisely in different farming settings, from indoor tanks to open-ocean cages. This article reviews what has been achieved in smart feeding technology and equipment so far, discusses the challenges that still limit their use on real farms—such as high costs, equipment breakdowns, and the need for trained workers—and suggests what should be improved next. The findings may help farmers and industry professionals understand how precision feeding works and how it can make aquaculture more efficient, environmentally friendly, and profitable.

## 1. Introduction

Aquaculture plays a vital role in global food security by supplying high-quality animal protein, supporting the fishery economy, and driving the transformation and upgrading of modern agriculture [1].

As a core management practice in aquaculture production, feeding directly affects the growth rate of cultured organisms, feed utilization efficiency, economic returns, and water-quality [2,3]. Insufficient feeding may inhibit normal growth and reduce size uniformity, whereas excessive feeding can lead to uneaten feed accumulation, water quality deterioration, and feed waste [2,3]. Therefore, achieving scientific, appropriate, and efficient feeding according to the actual requirements of cultured organisms has long been a key issue in aquaculture research and production [4,5,6].

For a long time, aquaculture feeding has mainly relied on human experience and fixed rules, and has generally been conducted at predetermined times and amounts. Related equipment has also been dominated by simple automatic feeders [7]. These methods are applicable to some extent in small-scale systems or relatively stable environments. However, in practical production, feeding requirements vary significantly among species, growth stages, and farming modes, making accurate matching difficult when relying solely on experience. In addition, environmental factors such as water temperature, dissolved oxygen, pH, weather, and season jointly affect feeding behavior and metabolism. As a result, the feeding process exhibits nonlinear, time-varying, and multifactorial characteristics. Driven by advances in machine vision, the Internet of Things, machine learning, deep learning, and automatic control, aquaculture feeding is shifting from experience-driven management to data-driven regulation, and from extensive management to precision control [4,5].

Current research on precision feeding in aquaculture can be broadly divided into two main directions. The first focuses on precision feeding technologies, including aquaculture status perception, feeding decision generation, and precise execution control. Its core objective is to address when to feed, how much to feed, where to feed, and how to dynamically adjust the feeding process [8,9]. The second focuses on precision feeding equipment for engineering implementation and system integration in different aquaculture scenarios. This direction covers fixed, rail-guided, pneumatic, unmanned-vessel-based, and platform-based systems, aiming to improve the reliability, applicability, automation level, and scalability of feeding systems [10]. Recent studies reviewing key precision feeding technologies and sensor-based precision feeding systems further indicate that precision feeding is increasingly developing toward integrated sensing, individualized decision support, and adaptive execution [11,12,13].

In recent years, extensive studies have been conducted along these two directions. At the technical level, key advances have been made in feeding demand perception, intelligent feeding decision-making, and precise execution control, providing important methodological support for precision feeding. At the equipment level, feeding devices and operating platforms suitable for different aquaculture modes have also developed rapidly, gradually evolving from single mechanical structures into intelligent systems integrating perception, decision-making, execution, and management. However, existing studies still face several limitations, including insufficient sensing robustness, limited model generalization, inadequate closed-loop coordination, equipment reliability issues and a lack of standardization. These issues restrict the further deployment and scalability of precision feeding technologies and equipment [14].

**Figure 1:**
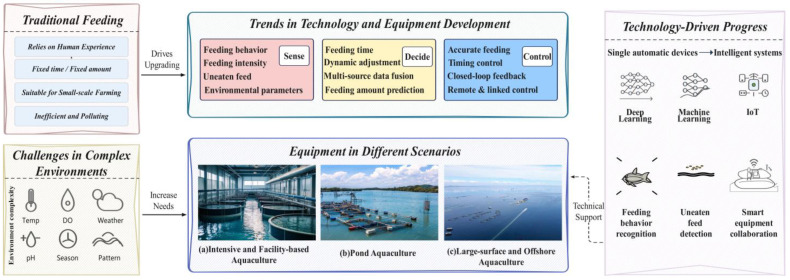
Overall framework for precision feeding technologies and equipment in aquaculture. Traditional feeding limitations and complex environmental conditions increase the demand for technological upgrading. The three horizontally arranged modules represent the main workflow of precision feeding, including feeding demand sensing, feeding decision-making, and precise control and execution. Solid arrows indicate the main direction of technological development and information transfer, whereas dashed arrows indicate the support and feedback relationships between technical modules and equipment applied in different aquaculture scenarios. DO denotes dissolved oxygen. (Self-produced).

Based on this context, the present study systematically reviews the research progress of precision feeding technologies and equipment in aquaculture from both technical and equipment-oriented perspectives. The technology section focuses on key technologies, including feeding demand perception, feeding decision generation, and precise execution control. The equipment section summarizes the main types, performance characteristics, and application progress of feeding equipment in different aquaculture scenarios. On this basis, the major challenges in existing research are analyzed, and future development directions are discussed, with the aim of providing a reference for research and engineering applications of precision feeding systems in aquaculture. The overall framework is shown in Figure 1.

Compared with previous reviews that mainly focus on either feeding technologies or feeding equipment separately, the novelty and added value of the present study lie in its integrated analysis of precision feeding from both technological and equipment-oriented perspectives. Specifically, the proposed framework connects feeding demand perception, feeding decision-making, and precise execution control with different types of feeding equipment used in intensive aquaculture, pond aquaculture, and large-scale or offshore aquaculture scenarios. This scenario-oriented synthesis helps clarify how sensing methods, feeding algorithms, control strategies, and equipment platforms can be coordinated to meet the specific requirements of different aquaculture systems. Therefore, the present study not only summarizes the current research status of precision feeding technologies and equipment, but also provides a systematic reference for the future development of adaptive feeding algorithms, closed-loop feeding systems, and scenario-specific intelligent feeding equipment in aquaculture.

## 2. Methods

To characterize research trends in precision feeding for aquaculture, a bibliometric search was conducted using the Web of Science Core Collection with no language restrictions. The overall search combined aquaculture-related terms with feeding-related terms, and seven supplementary searches were subsequently conducted within the overall dataset to identify publications corresponding to seven thematic categories. The complete Boolean search strings, search parameters, and corresponding record counts are presented in Table 1. Records unrelated to aquaculture feeding technologies or equipment were excluded after title and abstract screening.

**Figure 2:**
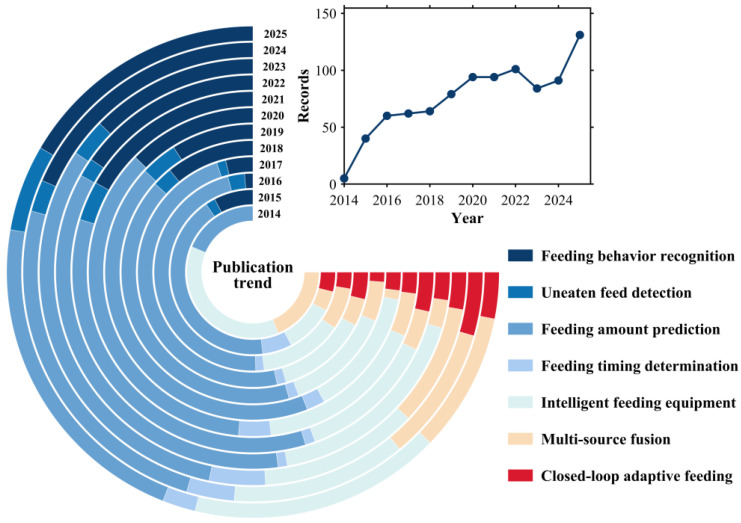
Annual and thematic research trends in precision feeding technologies and equipment in aquaculture from 2014 to 2025 (Self-produced).

As shown in Figure 2, research on precision feeding technologies and equipment in aquaculture showed a steady upward trend from 2014 to 2025 based on Web of Science Core Collection records. The overall annual number of publications increased from 5 in 2014 to 131 in 2025, indicating that precision feeding has become an increasingly important topic in intelligent aquaculture. From the perspective of thematic evolution, feeding amount prediction and intelligent feeding equipment have maintained relatively high publication levels over the past decade, reflecting their central roles in this field. In recent years, feeding behavior recognition, multi-source fusion, and closed-loop adaptive feeding have also shown increasingly active development, suggesting that the field is gradually evolving from conventional automated feeding toward integrated perception, intelligent decision-making, and feedback-driven control.

## 3. Framework of Precision Feeding in Aquaculture

### 3.1. Definition and Connotation of Precision Feeding

Precision feeding refers to the rational regulation of feeding amount, feeding timing, feeding location, and feeding frequency based on the dynamic the growth requirements, feeding status, and environmental conditions of cultured organisms. Its purpose is to achieve an effective match between feed supply and actual feeding demand. The main objectives of precision feeding are to improve feed utilization efficiency, reduce the accumulation of uneaten-feed and water pollution, and enhance aquaculture profitability and production management. Compared with traditional feeding methods that mainly rely on human experience or fixed rules, precision feeding places greater emphasis on the real-time acquisition and utilization of information related to the status of cultured organisms and the farming environment. Compared with automatic feeding methods that perform feeding according to fixed time and quantity, precision feeding further emphasizes the dynamic response to changes in feeding behavior, uneaten feed status, and environmental parameters.

In essence, precision feeding is not the simple application of a single technology or a single piece of equipment. Instead, it is a systematic process integrating status perception, feeding decision-making, precise execution, and feedback optimization. Its core lies in establishing a refined and adaptive feeding management approach oriented toward the actual requirements of cultured organisms.

### 3.2. Core Workflow of Precision Feeding Systems

A precision feeding system consists of three basic modules: status perception, feeding decision-making, and precise execution. First, the system acquires information on the feeding status of cultured organisms and environmental parameters through behavior recognition, uneaten feed detection, and water-quality monitoring, providing the foundational data for subsequent regulation. Second, based on the perception results, the growth requirements of cultured organisms, environmental changes, and historical feeding information, the system determines the appropriate feeding amount, feeding timing, and feeding area. Finally, precise execution of the feeding task is achieved through quantitative feed discharge, spreading control, and motion control of the operating platform. This process embodies the systematic operating logic of precision feeding, progressing from information acquisition to analysis, execution, and feedback regulation. It also provides an important basis for subsequent research on related technologies and equipment [6].

### 3.3. Application Scenarios and Species Differences

The implementation of precision feeding exhibits clear scenario-specific and species-specific differences. From the perspective of application scenarios, recirculating aquaculture systems, pond aquaculture, and large-scale open-water aquaculture differ substantially in spatial scale, environmental stability, infrastructure conditions, and operating modes. Consequently, the requirements for sensing methods, decision-making models, and feeding equipment vary accordingly. Factory-based aquaculture environments are relatively controllable and are therefore more suitable for the deployment of fixed feeding devices with a high degree of system integration. In contrast, pond and large-scale open-water aquaculture environments are more open and are susceptible to weather conditions, water disturbances, and operating range. These environments therefore impose higher requirements on mobile operating platforms, autonomous navigation, and remote management capabilities.

From the perspective of cultured organisms, fish, shrimp, and crabs differ markedly in feeding behavior, preferred water depth, feeding rhythms, and spatial distribution characteristics. These differences indicate that demand perception, feeding strategies, and equipment design should be tailored to specific cultured organisms rather than following a uniform approach [15]. Therefore, the research and application of precision feeding systems should be tailored to specific aquaculture scenarios and cultured organisms to improve technical applicability and system performance.

## 4. Precision Feeding Technologies in Aquaculture

Having clarified the concept, core workflow, and application-scenario differences in precision feeding, the central question can be distilled as follows: how can relevant technologies effectively support the feeding process? In general, precision feeding technologies focus on three aspects: aquaculture status acquisition, feeding strategy generation, and feeding process implementation. Their objective is to transform the feeding requirements of cultured organisms, environmental changes, and operational demands into executable and optimizable feeding schemes.

**Figure 3:**
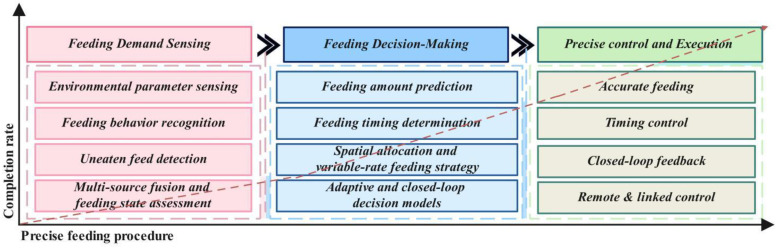
Implementation process of precision feeding technologies in aquaculture. The system progresses from feeding demand sensing to feeding decision-making and then to precise control and execution. The red dashed arrow indicates the increasing degree of process completion as sensing information is progressively transformed into executable feeding actions (Self-produced).

Among these three, demand perception is the prerequisite for system operation and determines the effectiveness of subsequent decision-making and control. Decision-making models form the core of the system generating the appropriate feeding amount, feeding timing, and feeding area. Execution and control technologies provide the necessary guarantee for precision feeding and directly affect the operational accuracy and application performance of the system. Therefore, this section systematically reviews the research progress of key technologies for precision feeding in aquaculture from three perspectives: feeding demand perception, feeding decision-making, and precise control and execution. The overall framework is shown in Figure 3.

### 4.1. Feeding Demand Sensing Technology

Feeding demand perception forms the foundation of precision feeding systems. Its main task is to acquire key information reflecting the feeding status of cultured organisms and changes in the aquaculture environment, thereby providing a basis for subsequent feeding decision-making and execution control. Unlike traditional feeding management based mainly on manual observation, current sensing systems increasingly integrate multiple types of information, including environmental parameters, feeding behavior, and uneaten-feed status. These correspond to different stages of the feeding process. Environmental parameters describe the external conditions affecting feeding activity, behavioral features indicate the immediate responses of cultured organisms, and uneaten feed provides direct feedback on feeding outcomes. Related research has mainly focused on environmental parameter monitoring, feeding behavior recognition, uneaten feed detection, and status assessment based on multi-source information fusion [16].

#### 4.1.1. Environmental Parameter Sensing

Environmental parameter monitoring refers to the acquisition and analysis of external conditions that affect the feeding behavior and metabolic processes of cultured organisms, including water temperature, dissolved oxygen, pH, ammonia nitrogen, and weather conditions. These variables provide essential contextual information for feeding regulation because aquatic animals respond sensitively to changes in their surrounding environment. Fluctuations in water temperature, dissolved oxygen, and water-quality indicators can alter the activity intensity, metabolic level, and feeding willingness of cultured organisms, thereby affecting the rational determination of feeding amount, feeding timing, and feeding area [17,18]. Feeding strategies based solely on fixed schedules or empirical rules cannot adequately accommodate dynamic environmental changes.

**Figure 4:**
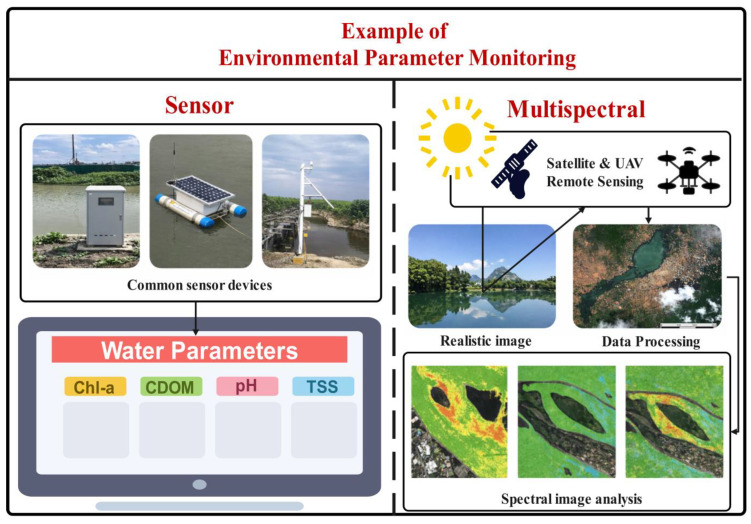
Environmental parameter monitoring based on sensor, UAV, and remote sensing methods (Self-produced).

Environmental monitoring methods differ mainly in spatial coverage, temporal continuity, and deployment conditions. Fixed-point online monitoring relies on multi-parameter water-quality sensors to continuously record temperature, dissolved oxygen, pH, and nutrient levels. This method is suitable for aquaculture systems with relatively stable conditions and well-defined monitoring locations [19]. Its main advantage lies in providing continuous local measurements, although a limited number of sensors may not fully represent spatial variation across larger ponds or open-water environments. Remote sensing and hyperspectral monitoring provide a complementary approach. Satellites, unmanned aerial vehicles, and hyperspectral imaging systems can be used to retrieve water-quality parameters over larger areas or at higher spatial resolution [20]. These methods improve spatial coverage but depend more strongly on image quality, retrieval-model accuracy, and environmental conditions during data collection. The basic workflow is shown in Figure 4.

**Figure 5:**
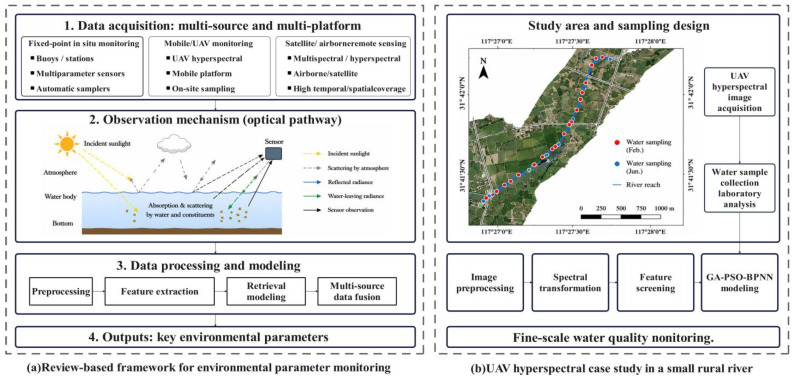
UAV hyperspectral monitoring and water-quality parameter retrieval in small rural rivers. Note: The figure was compiled and adapted by the authors based on Refs. [19,20].

Representative studies illustrate the different roles of these monitoring approaches. Chen [18] systematically reviewed the application of remote sensing technologies in inland water-quality monitoring and summarized the selection of data sources, index construction methods, and retrieval models. The study highlighted the value of remote sensing for long-term and large-area monitoring, while also emphasizing the need for multi-source data fusion and improved retrieval accuracy. Although the review focused primarily on inland water environments, its methodological framework is relevant to environmental sensing in precision aquaculture. Wang et al. [19] retrieved turbidity, ammonia nitrogen, total nitrogen, and total phosphorus in small rural rivers using UAV hyperspectral imagery and machine-learning methods and developed a GA-PSO-BPNN model. This study demonstrates that UAV-based hyperspectral monitoring can complement fixed-point sensors by providing fine-scale spatial information, especially when environmental conditions vary across the monitored area. The relevant research content is shown in Figure 5.

Overall, environmental parameter monitoring has gradually evolved from single-point and static data acquisition toward online, mobile, and remote sensing-based monitoring, providing essential environmental inputs for precision feeding. However, existing studies still have two major limitations. First, the relationship between environmental parameters and actual feeding demand remains complex, and unified characterization methods are still lacking for different species and aquaculture scenarios. Second, long-term operation is constrained by sensor drift, the limited representativeness of local measurements, and the dependence of high-accuracy retrieval models on high-quality data [21]. To address these issues, future research should further investigate the relationships between environmental parameters and feeding status and promote the coordinated deployment of fixed-point, mobile, and remote sensing monitoring methods.

#### 4.1.2. Feeding Behavior Recognition

Feeding behavior recognition refers to the assessment of feeding willingness and feeding activity by monitoring the external behavioral characteristics of cultured organisms during the feeding process, such as swimming, patterns, aggregation behavior, feeding competition, and activity intensity. Compared with environmental parameter monitoring, behavioral information can more directly reflect the immediate response of cultured organisms to feeding stimuli. Therefore, behavior recognition constitutes an important component of feeding demand perception [22,23].

Feeding demand is often first reflected in behavioral changes and subsequently manifested in feeding outcomes. Features such as aggregation degree, swimming speed, surface response, and food competition intensity serve as important indicators for evaluating appetite status and feeding demand. Accurate recognition of these behavioral features can provide more direct data support for feeding amount adjustment and feeding timing determination.

**Figure 6:**
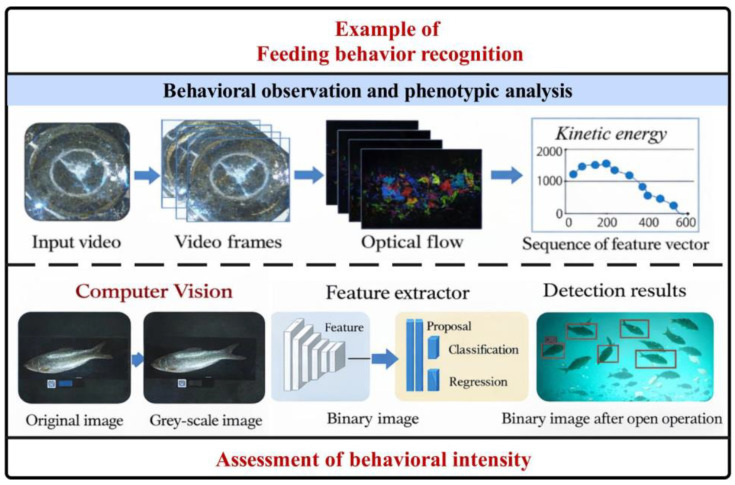
Behavioral observation and computer vision methods for feeding behavior recognition in aquaculture. Note: The figure was compiled and adapted by the authors based on Refs. [24,25,26].

Research in this area has developed along two complementary routes. The first is based on ethological observation and phenotypic analysis, which is mainly used to summarize the relationships among feeding, swimming, stress behaviors, and aquaculture management [24]. This approach provides an important biological basis for selecting behavioral indicators, but its reliance on manual observation limits real-time application. The second route relies on computer vision-based behavior recognition, in which image processing and deep learning models are used to extract activity features of fish schools and achieve automatic recognition of feeding behavior [25,26,27]. Compared with manual observation, vision-based methods improve the objectivity and temporal continuity of feeding-state assessment. The basic contents are shown in Figure 6. In addition, previous reviews have summarized vision-, sensor-, acoustic-, entropy- and fractal-based methods for fish feeding behavior assessment, suggesting that behavior recognition is moving from qualitative observation toward quantitative and welfare-oriented analysis [28,29].

**Figure 7:**
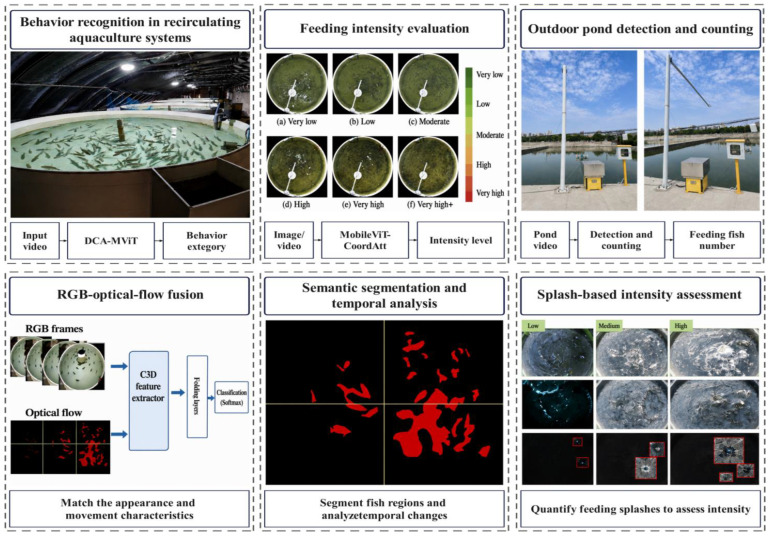
Representative studies on fish feeding behavior and appetite recognition in aquaculture. Note: The figure was compiled and adapted by the authors based on Refs. [32,33,34,35,36,37].

Representative studies illustrate the gradual expansion of behavior recognition from controlled aquaculture systems to more complex open environments. Hu et al. proposed the DCA-MVIT model, which improved the recognition performance of fish feeding behavior in recirculating aquaculture systems [30]. Liu et al. used the MobileViT-CoordAtt model to evaluate the feeding intensity of fish schools, enabling the shift from traditional experience-based judgment to quantifiable indicators [31]. Wang et al. estimated the number of feeding fish in outdoor ponds based on object detection and counting methods, extending behavior recognition technologies to open aquaculture scenarios [32]. These studies show that the research focus has shifted from identifying whether feeding behavior occurs to quantifying feeding intensity and estimating the number of actively feeding fish. Other recent studies have explored RGB-optical-flow fusion, semantic segmentation with temporal variance analysis, splash-based intensity assessment, and deformable-attention transformer models for hunger-degree identification, thereby expanding the available technical routes for appetite and feeding-state assessment [33,34]. The relevant research content is shown in Figure 7 [35,36,37].

The application scenarios, dataset sizes, and reported performance of representative feeding-behavior-recognition methods are summarized in Table 2.

As summarized in Table 2, the reported results should not be ranked solely by accuracy because the reviewed studies differ in cultured species, dataset size, recognition objective, application scenario, and evaluation metrics. Methods validated in controlled indoor environments may not maintain the same performance in outdoor ponds, where water turbidity, surface reflection, background interference, small targets, and fish occlusion are pronounced. Moreover, neither occlusion-specific metrics and inference latency are consistently reported, which limits a rigorous comparison of recognition robustness and real-time deployment capability. Future studies should adopt standardized evaluation metrics and conduct external validation under different aquaculture conditions.

Taken together, feeding behavior recognition has evolved from qualitative observation toward automated and quantifiable assessment. Ethological observation remains valuable for identifying biologically meaningful indicators, whereas computer vision and deep learning improve real-time monitoring capability and reduce dependence on subjective judgment. Nevertheless, the applicability of vision-based methods remains strongly scenario-dependent. Water turbidity, illumination variation, water-surface reflection, fish occlusion, and background complexity may reduce recognition reliability, particularly in outdoor ponds. Furthermore, variations in species, stocking density, camera position, dataset size, and evaluation metrics hinder direct comparison across existing models. Therefore, high recognition accuracy under a specific experimental setting does not necessarily guarantee stable performance across different aquaculture scenarios.

#### 4.1.3. Uneaten Feed Detection

Uneaten feed detection refers to the identification and analysis of feed particles that remain unconsumed after feeding. It is used to determine the feeding endpoint, assess the risk of overfeeding, and provide feedback for subsequent feeding adjustment. Compared with environmental parameter monitoring and feeding behavior recognition, uneaten-feed detection more directly reflect feeding outcomes and therefore holds strong practical value in feeding demand perception for precision feeding. Previous studies have shown that the accumulation of uneaten feed and feces can adversely affect the aquaculture water environment. Therefore, the timely identification of uneaten feed status is closely related not only to feeding accuracy but also to water-quality maintenance and aquaculture profitability [40,41,42,43].

**Figure 8:**
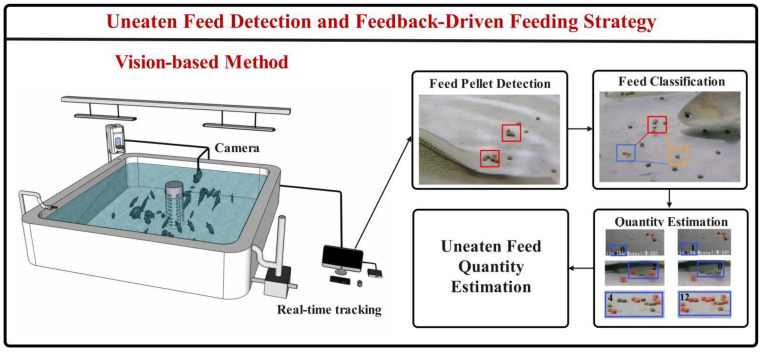
Vision-based uneaten feed detection and feedback-driven feeding strategy. Note: The figure was compiled and adapted by the authors based on Ref. [44].

If feeding demand is evaluated solely on the basis of pre-feeding behavior or environmental conditions, overfeeding and delayed or inaccurate termination of feeding may still occur. Uneaten-feed status directly reflect the degree of consistency between feeding outcomes and actual feeding demand, serving as an important basis for feeding endpoint determination and closed-loop correction. Compared with methods that rely solely on manual observation, machine vision- and image recognition-based uneaten feed detection improve the objectivity and real-time performance of feeding-status assessment. Current studies mainly use machine vision, object detection, and image recognition techniques to identify uneaten feed particles in real time. Unlike pre-feeding assessment methods, these approaches introduce feeding-outcome information into the regulation process and provide a basis for dynamic adjustment, as shown in Figure 8.

**Figure 9:**
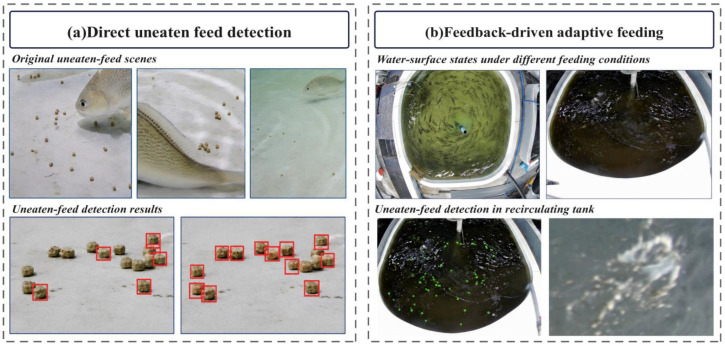
Representative studies on uneaten feed detection and adaptive feeding regulation in aquaculture. Note: The figure was compiled and adapted by the authors based on Refs. [44,45].

Representative studies demonstrate the transition from residual-pellet identification to feedback-based feeding regulation. Xu et al. proposed a real-time uneaten feed detection method based on an improved YOLOv5 model to address particle overlap, adhesion, long detection distance, and interference from fish bodies and water waves [44]. This method provides a direct technical approach for feeding endpoint determination and overfeeding identification. Li et al. further integrated uneaten feed detection with water-surface texture discrimination in an intelligent feeding strategies for factory-based recirculating aquaculture. By designing a test-feeding and multi-round adaptive feeding strategy within a single feeding cycle, this study showed that uneaten feed detection can be extended from a single recognition task to a feedback-driven feeding regulation process [45]. These studies indicate that the practical value of uneaten feed detection lies not only in recognizing residual particles but also in supporting timely feeding termination and adaptive adjustment. The relevant research content is shown in Figure 9.

Uneaten feed detection expands feeding demand perception from pre-feeding assessment to feeding-outcome feedback. Its engineering significance lies in whether residual-pellet information can be converted into timely control commands for reducing or terminating feed discharge. However, reliable application remains challenging because particle adhesion, particle occlusion, water-surface reflection, fish interference, and complex backgrounds may degrade detection accuracy. Differences in feed-pellet size, species, culture density, and farming environment also constrain the transferability of existing models. Therefore, future research should focus on integrating uneaten feed detection with feeding decision-making and execution control, while strengthening long-term validation under practical aquaculture conditions.

#### 4.1.4. Multi-Source Fusion and Feeding-State Assessment

Multi-source fusion and feeding status assessment refer to the integrated analysis of environmental parameters, feeding behavior, uneaten-feed status, and historical feeding information to form a higher-level judgment of the feeding demand. Compared with monitoring based on a single indicator, this approach can more comprehensively reflect changes in feeding status and is therefore better suited for feeding demand perception in complex aquaculture environments.

The necessity of multi-source fusion arises from the fact that feeding demand is usually jointly affected by multiple factors, including environmental conditions, behavioral responses, and feeding outcomes. Relying on a single type of information often cannot accurately characterize actual feeding status. For example, environmental parameters alone may not reflect immediate feeding willingness, whereas behavioral features alone may neglect feeding outcomes and water-quality constraints. Uneaten-feed information can directly indicate whether feed supply exceeds actual demand, but it does not fully explain the environmental or behavioral causes of the mismatch. Therefore, integrating multi-source information is an important direction for improving the accuracy and robustness of demand perception [46,47].

Current research involves two closely connected processes: multi-source data acquisition and integrated feeding-state assessment. At the data-acquisition level, visual information, water-quality measurements, acoustic signals, and platform-monitoring data can be combined to provide complementary descriptions of cultured organisms and their environment. At the assessment level, machine-learning models, neural networks, and rule-based methods process these heterogeneous inputs and generate higher-level outputs, such as feeding intensity, appetite level, and feeding demand. The first process improves information completeness, whereas the second determines whether heterogeneous data can be transformed into reliable and interpretable feeding decisions. Previous reviews have indicated that the application of machine learning in fish behavior analysis, water-quality prediction, and biomass estimation provides a methodological basis for integrated status assessment. For example, underwater stereo vision has been used to identify fish species and estimate body length automatically, providing biomass-related information to support more refined feeding decisions [48].

Representative studies show that multi-source fusion is gradually moving beyond status recognition toward direct support for feeding regulation. Tu et al. developed a dynamic feeding decision system for shrimp based on multi-source information fusion, integrating underwater visual monitoring, a two-factor decision model, PLC control, and host-computer management, thereby achieving dual regulation based on growth status and uneaten-feed feedback [49]. Similarly, Li et al. further combined water-surface texture discrimination with uneaten feed detection in an intelligent feeding strategy for factory-based recirculating aquaculture and implemented a test-feeding and multi-round adaptive feeding strategy within a single feeding cycle [45]. These studies indicate that multi-source information fusion has gradually extended from status recognition to feeding strategy correction and closed-loop optimization.

Multi-source fusion represents a shift from isolated monitoring toward integrated feeding-state assessment. Its value lies not in adding more sensors, but in combining complementary information and converting it into actionable feeding decisions. Nevertheless, the benefits of fusion depend on data quality, temporal synchronization, model complexity, and interpretability. Differences in sampling frequency, sensor accuracy, environmental interference, and data formats may compromise the reliability of integrated assessment. Moreover, overly complex models may improve recognition performance at the cost of higher computational requirements and reduced interpretability of the decision process. Therefore, the selection of a fusion strategy should balance information richness, real-time performance, interpretability, and deployment cost according to the cultured species and aquaculture scenario [50].

### 4.2. Feeding Decision-Making Technologies

**Figure 10:**
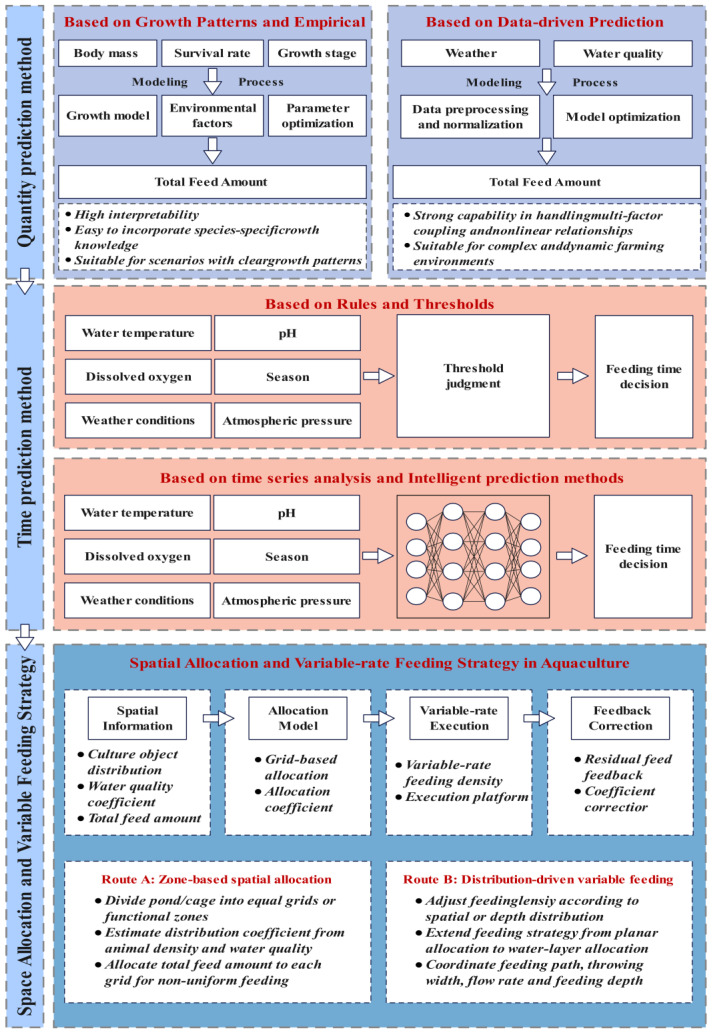
Feeding decision-making methods for precision feeding in aquaculture (Self-produced).

Feeding decision-making is the core component of a precision feeding system. Its main task is to transform feeding status, environmental information, and historical data into specific feeding schemes. Compared with traditional methods that rely on human experience to determine feeding amount and feeding timing, current studies place greater emphasis on quantitative decision-making based on data analysis, model calculation, and feedback information. The decision-making process can be analyzed from four perspectives: feeding amount prediction, feeding timing determination, spatial allocation and variable-rate feeding strategies, and feedback-based adaptive decision-making. These perspectives correspond to the determination of how much feed should be supplied, when feeding should be initiated or terminated, where feed should be distributed, and how feeding strategies should be dynamically adjusted during operation. The framework of this section is shown in Figure 10.

#### 4.2.1. Feeding Amount Prediction

Once feeding status has been perceived, translating environmental information, behavioral cues, and growth-related data into a specific feeding amount is the central task of precision feeding decision-making. Feeding amount prediction refers to the estimation of the amount of feed required within a certain period based on the current status of cultured organisms, environmental conditions, and historical production data. Its objective is to meet growth requirements while reducing feed waste and environmental load [51,52].

Feeding amount directly affects the regulation performance of a precision feeding system. Insufficient feeding can impair normal growth, whereas excessive feeding can lead to uneaten feed accumulation, water-quality deterioration, and increased production costs. Therefore, feeding amount prediction is not merely a simple quantitative calculation, but a core link connecting aquaculture demand, environmental constraints, and production efficiency. As aquaculture management gradually shifts from experience-based practice to data-driven regulation, feeding amount prediction has also evolved from traditional empirical estimation to quantitative analysis based on models and algorithms.

Existing methods differ mainly in their dependence on biological mechanisms and historical data. The first category comprises prediction methods based on growth patterns and empirical relationships. These methods typically estimate the total feeding amount by considering parameters such as body weight, survival rate, growth stage, and feeding rate. Such methods offer good interpretability and are suitable for integration with the growth patterns of specific cultured organisms. The second category encompasses data-driven prediction methods, in which water temperature, dissolved oxygen, ammonia nitrogen, weather conditions, stocking density, and historical feeding data are used as inputs, and machine learning or deep-learning models are applied to predict feeding amount. These methods offer distinct advantages in handling multifactorial effects and nonlinear relationships, and are more suitable for dynamic decision-making in complex aquaculture environments. A third category combines mechanistic models with data-driven algorithms, aiming to retain the interpretability of biological growth models while improving the ability to describe nonlinear environmental effects.

**Figure 11:**
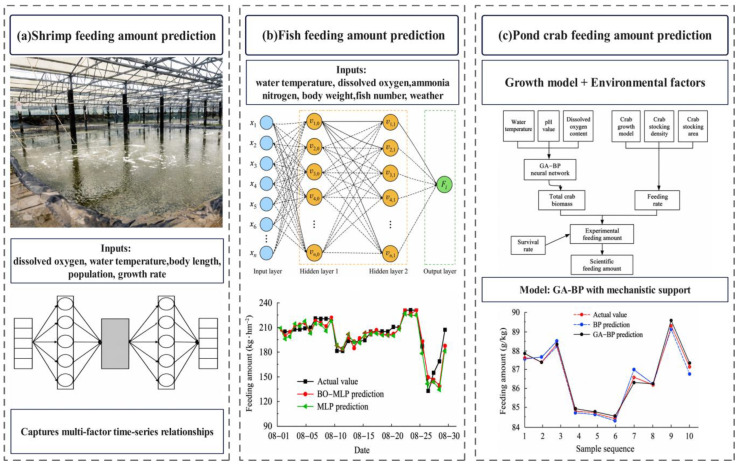
Representative feeding-amount-prediction methods in aquaculture. Note: The figure was compiled and adapted by the authors based on Refs. [53,54,55].

Representative studies illustrate the characteristics of these three approaches. Zhou et al. constructed a GA-LSTM-ATTN feeding amount prediction model [53] by integrating dissolved oxygen, water temperature, body length, stocking density, and growth rate as an additional variable. A genetic algorithm was used to optimize the LSTM parameters and the attention mechanism, improving its ability to represent multi-factor time-series relationships [53]. Zhu et al. developed a multilayer perceptron-based feeding amount prediction model using water temperature, dissolved oxygen, ammonia nitrogen, fish body weight, total fish number, and weather conditions as inputs, and adopted Bayesian optimization for hyperparameter tuning, demonstrating that the multilayer perceptron combined with optimization algorithms can support data-driven feeding amount prediction [54]. Unlike purely data-driven methods, Sun et al. investigated pond crab aquaculture from the perspective of a crab growth model, introduced environmental factors into a traditional biological growth model, and combined it with a GA-BP neural network to predict the total feeding amount. The high fitting accuracy achieved indicates that the integration of mechanistic models and data-driven models is also a feasible approach [55]. These studies indicate that no single model type is universally optimal. Deep-learning models are well suited for capturing temporal and nonlinear relationships, multilayer perceptrons provide a straightforward data-driven solution, and hybrid models offer a potential balance between predictive performance and biological interpretability. The relevant research content is shown in Figure 11.

The model characteristics, input variables, dataset sizes, and reported performance of representative feeding-amount-prediction methods are summarized in Table 3.

As summarized in Table 3, the three methods represent different modeling strategies and should not be treated as directly interchangeable alternatives. GA-LSTM-ATTN is suitable for describing temporal relationships among multiple variables, but its validation was based on 127 daily records collected from a single pond. BO-MLP was evaluated using multi-year data from multiple ponds and therefore provides stronger evidence of cross-pond generalizability under routine farming conditions. However, its prediction accuracy decreased during exceptional events, such as fish disease, acclimation, and large-scale harvesting. The growth-model-based GA-BP method incorporates biological-growth information and offers greater interpretability, but its broader generalizability remains uncertain because the reported prediction test included only 10 samples. These differences indicate that model selection should consider not only predictive accuracy, but also dataset size, biological interpretability, external validation, and adaptability to abnormal production conditions.

#### 4.2.2. Feeding Timing Determination

Feeding timing determination is necessary because the feeding activities of aquatic animals exhibit pronounced temporal variability. Under different environmental conditions, the appetite level and feeding rhythm of cultured organisms are not stable; conducting feeding solely at predetermined time points, is often inadequate for adapting to changes in actual feeding demand. Therefore, feeding timing determination is not merely a simple adjustment of a predetermined timetable, but an important component that incorporates environmental dynamics, behavioral responses, and historical patterns into the feeding decision-making process.

Existing methods differ mainly in the information used to trigger feeding operations. Traditional methods rely on empirical rules and predetermined schedules, they are easy to implement but cannot respond effectively to short-term changes in feeding demand. Environmental-threshold-based methods determine suitable feeding periods according to variations in water temperature, dissolved oxygen, or weather conditions, offering a simple structure convenient for practical production, but their performance depends strongly on the selection of threshold values. By contrast, time-series analysis and intelligent prediction models use multiple environmental variables and historical data to predict changes in feeding status, thereby generating more dynamic feeding timing decisions. Feedback-driven strategies further extend this process by adjusting feeding duration or termination timing according to real-time behavioral responses and uneaten-feed information.

Representative studies illustrate the transition from empirical timing determination to predictive and feedback-driven regulation. Han et al. proposed a feeding timing determination method for crab ponds based on water-quality parameter fusion and multivariate DeepAR, constructing a water-quality score from multiple water-quality parameters and predicting its future trajectory with multivariate DeepAR. The period corresponding to the peak score is then taken as the optimal feeding window, thereby transforming feeding timing selection from experience-based judgment to prediction based on environmental-status evolution [56]. Similarly, Li et al. combined water-surface texture discrimination with uneaten-feed feedback in an intelligent feeding strategy for factory-based recirculating aquaculture, and developed a test-feeding and multi-round adaptive feeding strategy within a single feeding cycle [45]. Unlike methods that determine only the initial feeding window, this strategy also adjusts the duration and termination of feeding according to real-time feedback. These studies indicate that feeding timing determination is evolving from static scheduling toward dynamic regulation before and during feeding operations.

Feeding timing determination has progressed from fixed schedules toward threshold-triggered, predictive, and feedback-driven regulation. These approaches serve different application requirements rather than forming a simple hierarchy. Schedule-based methods are easy to deploy; threshold-based methods offer limited adaptability at relatively low implementation complexity. Prediction models can identify suitable feeding windows by analyzing temporal trends; and feedback-driven methods are more suitable for adjusting feeding duration and termination timing during actual operations. Nevertheless, their transferability remains limited because feeding rhythms vary among species, growth stages, environmental conditions, and farming modes. Moreover, even accurate prediction alone cannot ensure effective operation if data acquisition, model calculation, and equipment response are not sufficiently timely. Therefore, the development of feeding timing determination methods should balance biological interpretability, real-time performance, and scenario adaptability.

#### 4.2.3. Spatial Allocation and Variable-Rate Feeding Strategy

Once feed amount and feeding timing have been determined, the feeding strategy must further address the spatial distribution of feed. Spatial allocation and variable-rate feeding tailor the location and intensity of feed delivery to the distribution of cultured organisms. By accounting for variation across areas, water layers, and time periods, these strategies improve feeding targeting and feed utilization efficiency.

Cultured organisms are often unevenly distributed in ponds, cages, and open-water environments. If uniform feeding is still applied across the entire area, local feed insufficiency and local overfeeding can occur simultaneously. This not only impairs feeding performance but also increases the risk of feed waste and water pollution. Spatial allocation and variable-rate feeding address this by refining the total feed supply into area-specific doses. This shift marks a key transition in precision feeding, moving from total-amount control to spatially refined regulation. Remote sensing and drone mapping have also been applied to quantify the spatial distribution of aquaculture biomass, demonstrating that spatial information can inform regionally differentiated feeding [57].

The design of spatial feeding strategies depends on the aquaculture scenario and the operational capability of the feeding platform. In pond aquaculture, regional division offers a practical approach to allocating feed across spatially heterogeneous areas. Aquaculture waters can be divided into grids or functional zones. Allocation coefficients are then calculated from the density of cultured organisms, water-quality parameters, and historical feeding data. In cages and other depth-dependent environments, spatial regulation must also account for the vertical distribution of cultured organisms. Feed density, delivery depth, and feeding paths are then adjusted across water layers. In open-water environments, variable-rate feeding depends on the positioning accuracy, navigation capability, coverage range, and endurance of mobile feeding platforms.

Representative studies chart the transition from planar regional allocation to three-dimensional feeding regulation. Sun et al. proposed a precision feeding system based on a growth model for pond crab aquaculture. After determining the total feeding amount, the system calculated area-specific allocation coefficients from crab distribution density and water-quality parameters in each pond zone. The total feed was then distributed across grid areas to achieve zone-based variable-rate feeding [55]. Their results showed that variable-rate feeding can effectively accommodates the uneven distribution of crabs and environmental heterogeneity within ponds. Extending this principle to the vertical dimension, Cui et al. designed a mobile autonomous feeding device capable of three-dimensional feeding at different underwater depths [58]. Related studies further confirmed that variable-rate feeding extends beyond planar allocation to differentiated feeding across water layers [59]. These studies demonstrate that spatial allocation must align with both the distribution characteristics of cultured organisms and the execution capability of the feeding platform.

Spatial allocation and variable-rate feeding extend precision feeding from total-amount prediction to scenario-specific feed distribution. Their practical value depends not only on the accuracy of regional allocation coefficients, but also on whether the feeding platform can execute the required feeding path, depth, and discharge rate. Existing studies remain confined to specific species and scenarios, and unified criteria for regional division and coefficient determination have yet to be established. Planar grid-based strategies, moreover, cannot be directly transferred to depth-dependent or large-scale open-water environments. Future development must therefore align biomass-distribution sensing, regional allocation models, and platform-execution capability with the demands of each aquaculture scenario.

#### 4.2.4. Adaptive and Closed-Loop Decision Models

As feeding amount prediction, feeding timing determination, and spatial allocation strategies mature, precision feeding decision-making is evolving beyond static scheme generation toward dynamic, real-time adjustment. Adaptive and closed-loop decision-making models involve the continuous incorporation of feedback information during the feeding process, including uneaten-feed status, behavioral responses, water-quality changes, and equipment operating status. These data are then used to revise the original feeding scheme in real time, aligning the consistency of feeding decisions more closely with actual demand.

A single feeding decision cannot reliably govern aquaculture production, because the feeding status of cultured organisms shifts continuously in response to environmental fluctuations and prior feeding outcomes. Fixed feeding strategies rarely satisfy the dynamic demands of complex aquaculture scenarios. Adaptive and closed-loop decision-making therefore improves the real-time responsiveness of feeding, rather than merely the real-time performance of individual decisions, and advances precision feeding from prediction-based to feedback-driven control.

Existing methods differ primarily in the type of feedback information used and the depth of strategy adjustment. A basic closed-loop approach adjusts feeding amount or feeding rhythm during a feeding cycle in response to uneaten-feed status, behavioral responses, or changes in water-surface texture—establishing a direct link between feeding outcomes and equipment control. By contrast, integrated adaptive models combine visual monitoring, water-quality parameters, historical feeding records, and equipment operating status. Rule-based reasoning, machine learning, and fuzzy decision-making then update the feeding strategy under multiple simultaneous factors. Compared with single-feedback correction, multi-source adaptive decision-making characterizes feeding status more comprehensively, yet imposes greater demands on data synchronization, model interpretability, and real-time computation. Recent pond aquaculture studies have introduced vision-language models and optical-flow-based behavior analysis to integrate biomass, water-quality, and feeding-behavior information for automated feeding decisions [60].

Adaptive and closed-loop decision-making marks the shift from static scheme generation to continuous in-operation correction. Single-feedback methods are relatively easy to implement and enable timely adjustment of feeding amount or termination timing. Multi-source adaptive models provide richer information and greater flexibility, but they also increase at the cost of greater system complexity and computational demands. Existing studies remain confined to specific species and scenarios, and the long-term stability of online updating mechanisms has not been fully verified under production conditions. The engineering application of closed-loop decision-making therefore requires a balance among feedback richness, against response speed, model interpretability, computational load, and equipment reliability.

### 4.3. Precise Control and Execution Technology

Precise control and execution technologies convert feeding decisions into physical operations, including quantitative feed discharge, spreading regulation, feeding-area adjustment, and dynamic in-operation correction. Compared with feed demand sensing and decision-making, the execution layer directly governs whether a calculated feeding strategy is implemented accurately and reliably. Current studies fall into two levels: actuator-level regulation, focusing on feeding motor and spreading-mechanism control, and system-level optimization, coordinating feed amount, environmental conditions, and growth objectives.

At the actuator level, conventional feedback control methods effectively regulate feed discharge rate, motor speed, and spreading intensity. PID-based control is straightforward to implement and imposes modest computational demands, making it suitable for embedded feeding devices. However, fixed PID parameters may prove insufficient when feed flow, motor load, environmental conditions, and feeding demand shift during operation. To improve adaptability, Zhao et al. combined fuzzy PID control with particle swarm optimization in a pond precision feeding system for grass carp. Dissolved oxygen, water temperature, and fish growth conditions were used as inputs, and a pond-scale comparative experiment was conducted to evaluate the system performance using dissolved oxygen, water temperature, and fish growth conditions as inputs; system performance was evaluated through a pond-scale comparative experiment [61]. Fuzzy logic has also been used to translate farming experience into feeding regulation. Soto-Zarazúa et al. developed a fuzzy-logic-based feeder system for intensive tilapia production, in which environmental variables such as water temperature and dissolved oxygen were used to determine appropriate feeding quantities [62]. These methods offer clear control logic and are readily deployable, yet their rule bases and parameter settings often require recalibration for different species and aquaculture scenarios.

Feedback-based execution closes the loop between feeding outcomes and machine regulation. Pradana and Horio proposed an automatic feeding control method based on feed-pellet counting and water-surface ripple characteristics [63]. The system estimated feeding activity from visual information and adjusted the feeding process accordingly. By directly coupling sensing with execution, this approach offers greater adaptivity than fixed-time and fixed-amount feeding. However, the reported validation was conducted primarily with real-environment video data and prototype-level experiments. Its long-term reliability under variable illumination, water-surface reflection, wind disturbance, and complex pond conditions has yet to be evaluated.

At the system level, model predictive control optimizes feeding strategies multiple under objectives and operational constraints. Chahid et al. compared several model predictive control formulations for fish-growth trajectory tracking in precision aquaculture [64]. They incorporated feeding rate, water temperature, and dissolved oxygen into the optimization and analyzed the trade-offs among growth tracking, feed conversion ratio, and production cost. This approach provides a systematic framework for variable-rate feeding and multi-objective regulation. However, its effectiveness hinges on the accuracy of the fish-growth model and the availability of reliable environmental data. Moreover, the reported results were based on numerical simulations rather than long-term production trials.

Reinforcement learning offers an alternative path toward adaptive feeding-policy optimization. Chahid et al. used Q-learning to optimize fish-growth trajectory tracking and feeding-rate control under changing environmental conditions [65]. Compared with fixed-rule control, reinforcement learning can update feeding policies through ongoing interaction with the system, making it well-suited to nonlinear and time-varying aquaculture processes. Nevertheless, most reinforcement-learning-based feeding studies remain at the simulation or laboratory stage. Their practical application remains constrained by training-data requirements, policy interpretability, safety constraints, and the need for stable performance under uncertain production conditions.

As summarized in Table 4, these control strategies serve distinct purposes and exhibit varying levels of engineering maturity. PID-based and fuzzy-control methods are well-suited for actuator-level implementation because of their low computational complexity and clear operating logic. Vision-based feedback control strengthens the connection between feeding outcomes and equipment regulation but remains susceptible to environmental interference. Model predictive control is suitable for multi-objective optimization when reliable dynamic models are available, whereas reinforcement learning offers greater adaptability to nonlinear and time-varying processes. Yet most studies on both approaches remain at the simulation or laboratory stage. Future precision feeding systems should adopt layered control architectures that combine reliable actuator-level regulation with feedback-based adjustment and system-level optimization. Greater attention should also be paid to distinguishing among numerical simulation, laboratory-scale validation, pond experiments, and long-term production applications.

## 5. Precision Feeding Equipment in Aquaculture

Precision feeding equipment is the primary vehicle through which precision feeding technologies are deployed in practice, and its maturity directly determines feeding performance and scalability. As aquaculture modes diversify, distinct scenarios impose specific demands on the structural forms design, operating mode, and functional integration of feeding equipment. Existing research on feeding equipment spans three principal scenarios: industrial and facility-based systems, pond aquaculture, and large-scale open-water and offshore operations. Feeding equipment is evolving from standalone feeding devices toward integrated systems that unify perception, decision-making, execution, and management. A recent review of intelligent and unmanned aquaculture equipment also confirms that feeding platforms, inspection robots, and autonomous operating systems are becoming the core infrastructure of smart aquaculture applications [66,67,68].

### 5.1. Equipment for Intensive and Facility-Based Aquaculture

Factory-based and facility-based systems are laid out with regular site layouts, well-developed infrastructure, stable power supply, and conditions favorable to equipment deployment. The aquaculture environment is relatively controllable, and the operating range and operating paths are well-defined. Accordingly, feeding equipment in these scenarios prioritizes fixed-point delivery, quantitative control, continuous and stable operation, and coordinated integration with monitoring and management systems [69].

**Figure 12:**
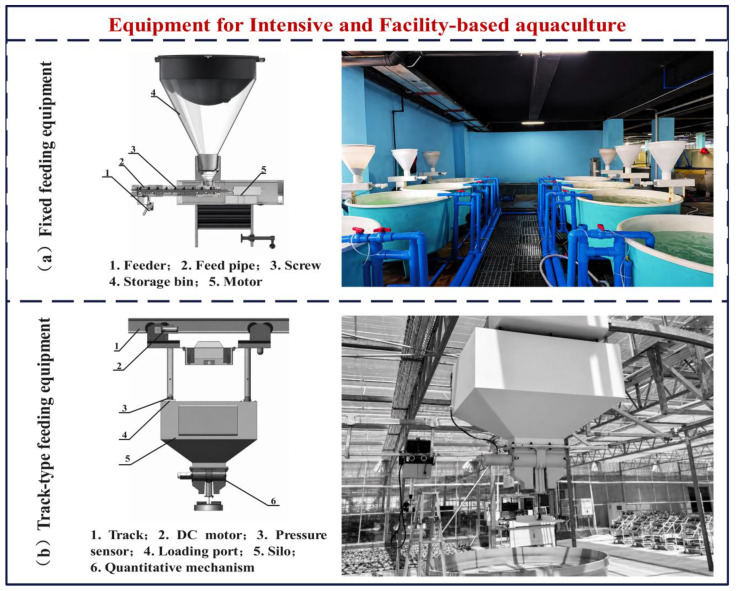
Representative feeding equipment for intensive and facility-based aquaculture. Note: The figure was compiled and adapted by the authors based on Refs. [70,71].

Based on these characteristics, feeding equipment falls into two categories. The first category is fixed or point-based feeding equipment, designed for settings where feeding locations are stable and the feeding process can be programmatically managed. Fully automatic precision feeding systems have demonstrated unmanned operation in large-scale aquaculture bases, substantially reducing labor costs and feed waste [70]. More recent systems incorporate real-time feedback that adjusts feeding speed in response to water-surface variations or feed competition signals during operation, allowing fixed equipment to evolve from open-loop execution toward feedback-regulated control. The second category is rail-guided and conveying-type feeding equipment, suited to scenarios with numerous aquaculture units, regularly distributed feeding points, and a requirement to improve coverage efficiency and automation level [71,72]. Common feeding equipment used in this scenario is shown in Figure 12.

Rail-guided precision feeding systems have integrated functions such as movement, weighing, feed discharge, and monitoring, with positioning errors controlled at the centimeter to millimeter level. This indicates their high engineering applicability in regularized aquaculture environments [73]. Rail-guided precision feeding robots designed for indoor factory-based aquaculture have further begun to adopt edge-cloud architectures and integrate data from UWB, IMU, and encoders to achieve unified positioning, navigation, and intelligent control. This indicates that rail-guided equipment is therefore developing toward higher precision, intelligence, and robotization.

### 5.2. Equipment for Pond Aquaculture

Pond aquaculture is characterized by open water, large operating ranges, pronounced environmental disturbance, and dispersed aquaculture units. The feeding process is susceptible to weather, water quality and the spatial distribution of cultured organisms. Compared with facility-based systems, pond aquaculture imposes greater demands on the mobility, operating coverage, path adaptability, and remote management capability of feeding equipment. Accordingly, pond feeding equipment relies primarily on mobile and platform-based systems, including feeding vehicles and feeding boats. These systems emphasize uniform and regionally differentiated feed distribution, together with autonomous operation, in open environments [74,75].

In pond aquaculture, earlier equipment consisted primarily of mobile feeding devices, aimed at reducing manual labor intensity and addressing concentrated feed delivery and uneven distribution. These devices are typically compact and moderately mobile, making them suited to local, fixed-point, or stratified feeding in small- and medium-scale ponds. As functionality has expanded, feeding equipment has evolved from simple surface feeding devices into multifunctional systems capable of three-dimensional feeding and auxiliary operations. As pond areas expand and operational demands increase, platform-based equipment, notably feeding vehicles and unmanned vessels has become a research focus. Operating from surface platforms, such equipment offers substantial feed-loading, cruising, and regionalized feeding capacity. By integrating feeding amount control, area-specific allocation, and platform motion capability, these systems better accommodate the uneven spatial distribution of cultured organisms in ponds [76,77]. In Chinese river crab farming, an automatic feeding boat integrating GPS/INS navigation, fuzzy PID control, full-coverage traversal, and bait distribution analysis was developed to improve feeding uniformity and reduce labor demand [78].

**Figure 13:**
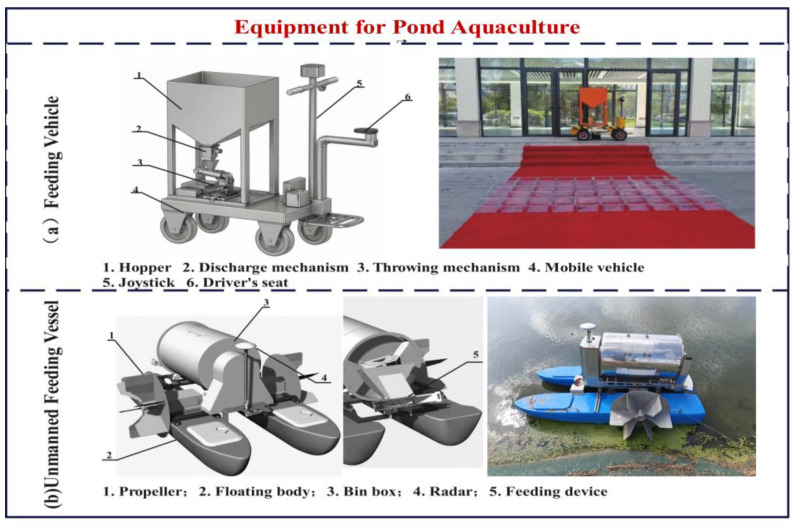
Representative feeding equipment for pond aquaculture. Note: The figure was compiled and adapted by the authors based on Refs. [80,81].

Pond feeding equipment is developing toward unmanned, systematic operation, with the research focus expanding from mechanical design alone to the integration of navigation and positioning, water-quality monitoring, remote scheduling, and operation management. These platforms therefore not only perform autonomous navigation and precision feeding, but also conduct environmental information acquisition, abnormality warning, and task management. This promotes the development of pond feeding from single-machine operation toward intelligent, networked, and coordinated operation [79]. Equipment research in pond scenarios also shows a trend toward integrated operations. Related platforms have begun to combine functions such as pesticide application, monitoring, and feeding. Equipment evaluation indicators have expanded from task completion to include feeding capacity, feeding width, operating accuracy, endurance, and operating cost. Pond feeding equipment is thus shifting from mechanized labor replacement toward precision operation and greater engineering applicability in open environments [80,81,82,83]. Common pond aquaculture equipment is shown in Figure 13.

Precision feeding equipment in pond aquaculture has developed into a diversified pattern spanning from mobile devices and water-surface platforms to integrated systems. The defining requirements across these forms are mobility, regionalized feeding capability, and autonomous operation. Future research should prioritize path adaptability in complex environments, continuous operation stability, environmental sensing coordination, and cost-effective application [84,85].

### 5.3. Equipment for Large-Surface and Offshore Aquaculture

Large-scale open-water and offshore aquaculture is characterized by large operating spaces, severe environmental disturbance, long feeding distances, and stringent continuous-operation requirements. Compared with pond and facility-based systems, these environments impose greater demands on the stability, loading capacity, long-distance conveying capability, and wind-wave resistance of feeding equipment. Feeding equipment in these scenarios therefore relies primarily on centralized feed supply, long-distance conveying, and platform-based operating systems, with emphasis on stable operation under large-scale, continuous, and high-intensity working conditions [86,87,88].

In open-water and offshore environments, earlier equipment consisted primarily of centralized feed supply and fixed-point feeding systems, aiming to address problems such as low efficiency of manual feed replenishment, limited feeding distance, and inconvenient feed supply for multiple cages after the expansion of aquaculture scale. They typically incorporate large feed storage units and fixed conveying lines, making it suitable for centralized feed supply and fixed-point distribution in regularly arranged aquaculture areas. With continued expansion, these systems have evolved from single-point feed supply to multi-point coordination and long-distance conveying modes to meet larger-scale operational demands.

Long-distance conveying equipment has become essential in large-scale open-water and offshore aquaculture [89]. These systems typically combine feed storage, conveying, and distribution functions, delivering feed reliably to multiple aquaculture units through pneumatic conveying, pipeline conveying, or mechanical conveying. This improves feed supply efficiency and reduces the frequency of manual transfer. Compared with mobile equipment used in pond scenarios, these systems place greater emphasis on continuous feed supply capability, conveying stability, and multi-point coordination. The research in these systems has shifted from the feeding mechanism to process control and continuous system operation reliability [90,91,92].

**Figure 14:**
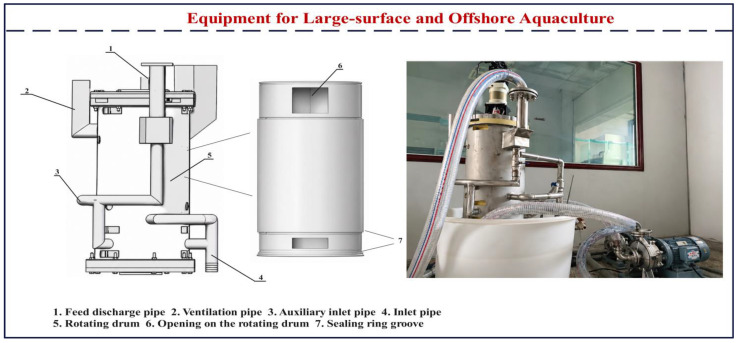
Representative feeding equipment for large-scale open-water and offshore aquaculture. Note: The figure was compiled and adapted by the authors based on Ref. [89].

As aquaculture environments, offshore feeding equipment is evolving toward platform-based and integrated systems. Research has moved beyond a single feeding mechanism, toward the coordinated integration of feed storage, conveying, delivery, monitoring, communication, and management functions. Such platforms can not only perform large-scale feed supply, but also provide environmental monitoring, operation management, and remote control. Since these platforms must typically operate for extended periods in wind-wave and corrosive environments, their design prioritizes on structural reliability, disturbance resistance, and maintenance convenience. Common offshore feeding equipment is shown in Figure 14.

Equipment research for large-scale open-water and offshore environments shows a clear shift toward system-level integration. Equipment evaluation has moved beyond task completion and extended to include feed storage capacity, conveying distance, feed supply stability, operational continuity, and platform adaptability. The development focus of such equipment is thus shifting from standalone feed supply capability toward stable system operation and full-scenario adaptability under complex environmental conditions.

Precision feeding equipment for large-scale open-water and offshore aquaculture has developed into a pattern spanning from centralized feed supply systems and long-distance conveying systems to platform-based integrated systems. These systems are defined by high loading capacity, long-distance reach, continuous-feed operation and disturbance tolerance. Future research should focus on adaptability to extreme environments, long-term operational reliability, fault diagnosis and maintenance convenience, and system-level integration.

### 5.4. Comparative Analysis of Representative Equipment

To compare the economic applicability of precision feeding equipment in different aquaculture scenarios, Table 5 summarizes the relative initial investment, operating cost, main cost drivers, and suitable production scale of representative equipment. Because equipment configuration, farm size, labor cost, energy price, and maintenance conditions vary widely among regions, the costs are evaluated qualitatively rather than as fixed monetary values.

As shown in Table 5, current precision feeding equipment for aquaculture spans fixed, rail-guided, mobile, vessel-mounted, unmanned-platform-based, and centralized feed supply systems. These equipment types differ markedly in applicable scenarios, operating modes, performance priorities and cost profiles. Because equipment configurations, farm scales, labor costs, energy prices, and maintenance conditions vary among regions, Table 5 presents relative cost levels rather than fixed monetary values.

In facility-based and factory-based aquaculture, representative equipment consists primarily of fixed integrated feeding systems, rail-guided feeding systems, and pneumatic feed supply systems. These systems are tied to regularized sites and well-developed infrastructure, and are characterized by stable quantitative feed discharge, predictable operation, and high system integration. They support continuous and standardized feeding, but their limited mobility and dependence on site infrastructure restrict their adaptability to open-water environments. Fixed automatic feeding systems typically incur low operating costs, whereas rail-guided and pneumatic systems involve moderate or moderately high costs owing to track maintenance, pipeline management, energy consumption, and sensor calibration requirements.

Equipment used in pond aquaculture is more diverse than that in facility-based systems, spanning mobile feeding vehicles, intelligent feeding boats, unmanned autonomous feeding vessels, and integrated unmanned platforms combined feeding and medication delivery. These systems emphasize mobility, regional coverage, and path adaptability in open environments. Small mobile devices are suitable for local and stratified feeding, but are limited in feed-loading capacity and endurance. Vessel-mounted and unmanned platforms provide a superior balance of loading capacity, coverage range, regionalized feeding, and continuous operation, and represent a major research direction for pond precision feeding. However, their operating costs rise as batteries, propulsion systems, positioning modules, communication devices, sensors, and software-management functions are integrated. Intelligent feeding boats therefore involve moderate operating costs, whereas highly integrated unmanned platforms require moderate-to-high or high operating expenditure.

By contrast, equipment for large-scale open-water and offshore aquaculture mainly includes centralized feed supply systems, long-distance conveying systems, and large platform-based feeding equipment. These systems offer substantial feed-loading capacity and sustained feed delivery, and are suited to large-scale operations. However, they entail more complex structures, higher construction and maintenance costs, and stricter requirements for operating environments and engineering support. Their main operating costs arise from conveying energy consumption, corrosion protection, pipeline maintenance, fault inspection, and specialized technical support. Although initial investment and operating costs are substantial, these systems can be economically advantageous at an appropriate production scale because of their high feed-loading capacity and continuous operation capability.

In performance terms, facility-based equipment prioritizes fixed-point quantitative control and operational stability. Pond platform-based equipment emphasizes regional matching and spatial adaptability; while large-scale open-water and offshore systems target long-distance conveying and continuous feed supply. From an economic perspective, equipment selection must also account for production scale, labor availability, equipment utilization rate, maintenance capacity, and long-term operating costs. Representative equipment is evolving from single feeding devices toward integrated systems that combine perception, decision-making, execution, and management, with clear trends toward modular design, platform-based integration, and intelligent operation.

Different equipment types exhibit no universal superiority, but rather distinct scenario adaptability. Future development must balance feeding accuracy, operating efficiency, continuous operation capability, system reliability, and life-cycle cost. It must also advance equipment evolution toward modularization, integrated platforms, and scenario-oriented adaptation [93,94].

## 6. Challenges

Although precision feeding in aquaculture has advanced in demand perception, feeding decision-making, precise execution, and equipment integration, it remains in a transitional stage from automatic to intelligent precision feeding. Its large-scale deployment in complex aquaculture therefore remains limited.

First, demand perception remains insufficiently robust. Visual recognition is susceptible to water turbidity, illumination variation, surface reflection, and target occlusion. Environmental monitoring faces sensor drift, fouling, and the limited representativeness of local measurements. Differences across species, growth stages, and farming modes compound this challenge by restricting the transferability of perception models. Future systems must therefore prioritize stable performance under changing environmental and operational conditions rather than focusing solely on short-term recognition accuracy [95,96].

Second, most feeding decision-making models are developed for specific species, environments, and limited datasets, resulting in weak cross-scenario generalization. Although some data-driven models achieve high prediction accuracy, their links to feeding mechanisms, growth patterns, and environmental constraints remain insufficient, which limits their interpretability and practical reliability. Integrating biological mechanisms with data-driven methods may improve model transferability [97,98].

Third, coordination among perception, decision-making, and execution remains inadequate. Many systems can recognize feeding behavior, monitor water quality, or detect uneaten feed, but the execution layer often still operates on fixed-time and fixed-amount control. Real-time feedback-based adjustment has yet to be fully realized. Delays in data transmission, decision generation, and actuator response may further degrade feeding accuracy. Abnormal-state detection, fault alarms, manual override functions, and fail-safe strategies are therefore necessary [99,100,101,102].

Fourth, long-term equipment reliability and standardization remain persistent and unresolved challenges. Feeding devices, sensors, communication modules, and mobile platforms frequently operate in humid, corrosive, and biologically active environments. Biofouling, corrosion, mechanical wear, power instability, and communication interruptions can compromise operational continuity and raise maintenance requirements. The absence of unified interfaces, communication protocols, data formats, and evaluation standards further hinders system integration and performance comparison [103].

Fifth, the adoption of precision feeding technologies varies considerably with economic conditions, infrastructure levels, and technical support capacity across regions. Many small-scale farms and resource-limited regions still depend on manual feeding, owing to restricted investment capacity, limited access to maintenance services, and a shortage of trained operators. In these contexts, farmers often prefer familiar manual feeding practices over unfamiliar automated systems, even where the latter offer long-term economic and environmental benefits.

Effective implementation depends not only on automated equipment but also on skilled personnel for sensor calibration, maintenance, interpretation of model outputs, and timely intervention. Operator training, after-sales support, manual override functions, and easy-to-maintain modular equipment are particularly important for small- and medium-scale farms. Economic feasibility should be evaluated from a life-cycle perspective. This includes initial investment, energy consumption, maintenance, component replacement, personnel training, production scale, equipment utilization rate, and potential reductions in labor demand and feed waste. Deploying precision feeding equipment without adequate human capacity building is unlikely to succeed. Ordinary farmers often lack the technical background to operate and maintain automated systems. Without accessible training programs and ongoing technical support, they tend to revert to familiar manual feeding methods. Therefore, the promotion of precision feeding must be matched by investment in operator education, skill development, and local technical service networks.

Finally, current research still lacks sufficient data and long-term validation. Public datasets are limited, and many studies remain at the stage of algorithm validation or prototype testing.

Quantitative reporting also lacks standardization. Existing studies employ different species, datasets, experimental conditions, validation procedures, and evaluation metrics, which makes direct aggregation difficult. Error bars, repeated-validation results, prediction horizons, and inference speed are not reported consistently. Improvements in feed conversion ratio attributable to specific sensing, decision-making, or execution technologies are rarely evaluated under comparable experimental designs. Benchmark trends over time are therefore difficult to establish, and realistic engineering targets remain unclear. Future studies should conduct multi-season, multi-species, and multi-region field trials and report standardized indicators. These should include uncertainty measures, feed conversion ratio, growth performance, feeding accuracy, inference speed, labor savings, energy consumption, equipment failure rate, and maintenance cost. Public datasets, unified benchmarks, and standardized evaluation procedures are essential for improving reproducibility and industrial applicability [104].

## 7. Conclusions and Future Perspectives

Precision feeding is a critical pathway toward efficient, sustainable, and intelligent aquaculture. This paper systematically reviews its system framework, key technologies, and representative equipment. Precision feeding has evolved into a technical system centered on demand perception, feeding decision-making, precise execution, and feedback optimization. Differentiated equipment pathways have emerged for factory-based recirculating aquaculture, pond aquaculture, and large-scale open-water and offshore aquaculture. Existing studies show that aquaculture feeding is shifting from experience-driven and rule-based control toward data-driven regulation, model-supported decision-making, and platform-based coordination. Equipment forms are evolving from single feeding devices to integrated and platform-based systems [44].

Future development should shift from refining individual technologies toward system-level coordinated optimization. Multi-source sensing fusion and feeding status assessment must be advanced to improve recognition robustness and cross-scenario adaptability in complex environments. Mechanistic models and data-driven methods should be integrated more deeply to enhance the generalization, interpretability, and online updating capability of decision-making models. Closed-loop coordination among perception, decision-making, and execution must be strengthened so that variable-rate feeding, regionalized feeding, and dynamic correction can move from local validation to system-level implementation. At the equipment level, priorities include modular design, low-cost deployment, long-term reliability, and multifunctional integration, along with unified standards for data, interfaces, and evaluation. As artificial intelligence, the Internet of Things, unmanned systems, and modern aquaculture practices become more deeply integrated, precision feeding is poised to become a key pillar for smart aquaculture. It will also play a growing role in improving profitability, reducing resource waste, and promoting sustainable development [1,4]. Within this framework, advances in machine-vision-based behavior recognition, deep-learning feeding decision models, IoT-based environmental sensing, and closed-loop adaptive control are expected to further strengthen the perception–decision–execution chain. Modular and integrated feeding equipment, in turn, can support reliable deployment across pond, RAS, and open-water aquaculture systems.

## Figures and Tables

**Table 1:** Search strategies used for the bibliometric analysis of precision feeding technologies and equipment in aquaculture. (Self-produced).

Search Set	Thematic Category	Boolean Search String	Current Record Count
#1	Overall precision feeding research	TS = ((“aquaculture” OR “fish farm*” OR “shrimp farm*” OR “recirculating aquaculture system*” OR “pond aquaculture” OR “cage culture” OR “offshore aquaculture” OR “mariculture” OR “shellfish culture” OR “crustacean culture” OR “intensive aquaculture”) AND (“feeding” OR “feed*” OR “precision feeding” OR “intelligent feeding” OR “automatic feeding” OR “smart feeding” OR “feeding management” OR “feeding strategy*” OR “feeding control” OR “feeding decision”))	905
#2	Feeding behavior recognition	#1 AND TS = (“feeding behavior” OR “feeding behaviour” OR “feeding activity” OR “feeding intensity” OR “appetite assessment” OR “fish aggregation” OR “schooling behavior” OR “feeding competition” OR “surface splashing”) AND TS = (“recognition” OR “detection” OR “classification” OR “computer vision” OR “deep learning” OR “machine vision” OR “image processing” OR “object detection” OR “behavior* analysis” OR “CNN” OR “YOLO”)	166
#3	Uneaten feed detection	#1 AND TS = (“uneaten feed” OR “residual feed” OR “unconsumed feed” OR “feed pellet*” OR “feed residue” OR “feed waste” OR “overfeeding” OR “excess feed”) AND TS = (“detection” OR “recognition” OR “identification” OR “YOLO” OR “object detection” OR “image recognition” OR “machine vision” OR “deep learning”)	37
#4	Feeding amount prediction	#1 AND TS = (“feeding amount” OR “feeding rate” OR “feed quantity” OR “feeding quantity” OR “feed intake” OR “feed requirement” OR “daily feed” OR “feed allowance” OR “feed conversion”) AND TS = (“prediction” OR “prediction model” OR “estimation” OR “forecast*” OR “machine learning” OR “deep learning” OR “neural network” OR “regression” OR “time series” OR “LSTM” OR “data-driven”)	429
#5	Feeding timing determination	#1 AND TS = (“feeding timing” OR “feeding time” OR “feeding frequency” OR “feeding schedule” OR “feeding window” OR “optimal feeding time” OR “feeding rhythm” OR “feeding period”) AND TS = (“optimization” OR “optimal” OR “decision” OR “strategy” OR “algorithm” OR “determination” OR “prediction” OR “scheduling”)	30
#6	Intelligent feeding equipment	#1 AND TS = (“feeding equipment” OR “automatic feeder” OR “feeding system” OR “feeding machine” OR “feeding robot” OR “feeding vessel” OR “feeding boat” OR “feeding platform” OR “intelligent feeding” OR “automated feeding” OR “feeding device” OR “feeding barge” OR “feeding ship” OR “rail-guided feeding” OR “pneumatic feeding”)	182
#7	Multi-source fusion	#1 AND TS = (“multi-source fusion” OR “multi-sensor fusion” OR “information fusion” OR “data fusion” OR “multimodal fusion” OR “sensor fusion” OR “multi-parameter fusion” OR “integrated sensing” OR “multi-modal perception”)	78
#8	Closed-loop adaptive feeding	#1 AND TS = (“closed-loop” OR “feedback control” OR “adaptive feeding” OR “adaptive control” OR “real-time adjustment” OR “dynamic feeding” OR “feedback-driven” OR “self-adaptive” OR “feedback-based feeding” OR “online adjustment”)	38

Notes: The Topic (TS) field covers title, abstract, author keywords, and Keywords Plus. The seven thematic categories (#2–#8) are non-exclusive; a publication covering multiple themes may be assigned to more than one category. Records unrelated to aquaculture feeding technologies or equipment were excluded after title and abstract screening.

**Table 2:** Quantitative comparison of representative feeding-behavior-recognition methods in aquaculture (Self-produced).

Ref.	Model or Method	Species and Application Scenario	Dataset Size	Reported Metric and Performance
[30]	DCA-MVIT	Perch (Lateolabrax japonicus) feeding-behavior recognition in a recirculating aquaculture system	16 videos containing 97,740 raw frames; final dataset: 5090 training images, 326 validation images, and 326 test images	Precision: 96.62%; Top-1 accuracy: 83.13%; mean F1-score: 83.98%; time-series video accuracy: 88.05%
[32]	YOLOv8-FishDetect based on object detection and counting	Outdoor pond aquaculture; mainly grass carp, with a small number of bighead carp and crucian carp	1273 images collected over five days	Precision: 84.6%; recall: 83.1%; mAP@0.5: 89.8%; mAP@0.5–0.95: 39.0%
[38]	ECA-DeepLabv3+ combined with FAIvar temporal variance analysis	Fish-school feeding-behavior recognition in indoor aquaculture tanks	1400 annotated frames from two datasets, including 900 images from DLOUSegDataset and 500 images from Cui’s dataset; 100 videos were randomly selected for behavior-recognition verification	Feeding-behavior-recognition accuracy: 90%; segmentation mIoU: 93.61%; segmentation mAcc: 94.97%
[39]	Semi-supervised feeding-splash detection and regression multiclassification	Feeding-intensity assessment for American black bass (Micropterus salmoides) based on pond-surface splashes	31,000 images in total, including 3100 labelled images and 27,900 unlabelled images	Mean accuracy: 98.22%; false detection rate: 1.27%; missed detection rate: 2.75%; splash-detection mAP: 85.11%

Note: DCA-MVIT, DSGated convolution and Coordinate Attention for Multiscale Vision Transformers version 2; CAA, Context Anchor Attention; DyHead, Dynamic Detection Head; ECA, Efficient Channel Attention; CAM, Class Activation Map; ACC, accuracy; mAP, mean average precision; mIoU, mean intersection over union; mAcc, mean accuracy; FPR, false positive rate; FNR, false negative rate; NR, not reported. Higher ACC, precision, recall, F1-score, mAP, mIoU, and mAcc values indicate better performance, whereas lower FPR and FNR values are preferable. The reported metrics are not directly comparable because the studies differ in recognition objectives, species, datasets, and application scenarios.

**Table 3:** Quantitative comparison of representative feeding-amount-prediction methods in aquaculture (Self-produced).

Ref.	Model or Method	Species and Application Scenario	Dataset Size	Reported Metric and Performance
[53]	GA-LSTM-ATTN	Pacific white shrimp (Penaeus vannamei) aquaculture in an outdoor pond	127 daily records collected over 127 days; training, validation, and test sets were divided at a ratio of 65:15:20	R^2^ = 0.8683; RMSE = 0.3703; MAE = 0.3311
[54]	BO-MLP	Catfish pond aquaculture	2760 daily records from six ponds and multiple years, including 1656 training samples, 552 validation samples, and 552 test samples	For Pond 9# in August, MAPE = 4.12%; for Pond 11# during the peak feeding period in August and September, MAPE remained below 4%
[55]	Growth-model-based GA-BP neural network analysis	Pond crab aquaculture	The total training-sample size was not explicitly reported; leave-one-out training and 10 test samples were used for model evaluation	R^2^ = 0.990; MSE = 0.04075 kg^2^; RMSE = 0.20196 kg

Note: R^2^, coefficient of determination; RMSE, root mean square error; MAE, mean absolute error; MSE, mean square error; MAPE, mean absolute percentage error; NR, not reported. Higher R^2^ values indicate better model fitting, whereas lower RMSE, MAE, MSE, and MAPE values indicate better predictive performance. The reported metrics are not directly comparable because the reviewed studies differ in cultured species, dataset size, input variables, validation conditions, and evaluation metrics.

**Table 4:** Critical comparison of representative control and execution methods for precision feeding in aquaculture (Self-produced).

Control Method	Main Control Objective	Representative Application	Validation Level	Main Advantage	Main Limitation
Fuzzy PID with optimization	Feed discharge regulation	Pond precision feeding for grass carp [61]	Pond-scale comparative experiment	Practical implementation and adaptive parameter adjustment	Requires scenario-specific tuning
Fuzzy logic control	Feeding-quantity determination	Intensive tilapia feeding system [62]	Controlled aquaculture experiment	Clear rule base and easy integration of farming experience	Limited transferability across species
Vision-based feedback control	Dynamic feeding adjustment	Pellet counting and ripple-feature analysis [63]	Real-environment video and prototype validation	Direct connection between sensing and execution	Sensitive to environmental interference
Model predictive control	Growth tracking and variable-rate feeding	Optimization of feeding rate, temperature, and dissolved oxygen [64]	Numerical simulation	Multi-objective optimization and constraint handling	Depends on model accuracy
Reinforcement learning	Adaptive feeding-policy optimization	Q-learning-based fish-growth trajectory tracking [65]	Numerical simulation	Adaptability to nonlinear and time-varying processes	Limited production validation and low interpretability

**Table 5:** Cost-oriented comparison of representative precision feeding equipment in different aquaculture scenarios (Self-produced).

Representative Equipment	Application Scenario	Relative Initial Investment	Relative Operating Cost	Main Operating Cost Drivers	Suitable Scale
Fixed automatic feeding system	Intensive and facility-based aquaculture	Low	Low	Electricity, cleaning, and routine maintenance	Small to medium
Rail-mounted precision feeding system	Intensive and facility-based aquaculture	Moderate	Moderate	Track maintenance, electricity, and sensor calibration	Medium to large
Pneumatic feeding system	Facility-based or pond aquaculture	Moderate	Moderate to high	Blower energy consumption, pipeline maintenance, and blockage removal	Medium to large
Mobile feeding vehicle	Pond aquaculture	Low	Low	Fuel or electricity and routine maintenance	Small to medium
Intelligent feeding boat	Pond aquaculture	Moderate	Moderate	Battery charging, propulsion maintenance, and positioning calibration	Medium
Unmanned autonomous feeding boat	Pond aquaculture	Moderate to high	Moderate to high	Battery replacement, sensor calibration, communication, and software management	Medium to large
Integrated unmanned platform	Pond aquaculture	High	High	Multi-module maintenance, sensor replacement, and operator training	Medium to large
Centralized feeding and conveying system	Large-surface and offshore aquaculture	High	High	Conveying energy consumption, corrosion protection, pipeline maintenance, and technical support	Large

Note: Relative cost levels are qualitatively classified as low, moderate, moderate to high, or high according to system complexity, energy consumption, maintenance requirements, component replacement, technical-support requirements, and personnel input. Direct monetary comparison is difficult because equipment configuration, farm scale, labor cost, energy price, and reporting methods vary among regions and studies.

## Data Availability

No new data were created or analyzed in this study. This article is a review of previously published literature, and the bibliometric data were derived from the Web of Science Core Collection, a publicly accessible database. The complete search strategies and Boolean search strings are described in Section 2. Data sharing is not applicable to this article.
